# Tibiotalocalcaneal (TTC) arthrodesis with reverse PHILOS plate and medial cannulated screws with lateral approach

**DOI:** 10.1186/s12891-017-1666-2

**Published:** 2017-07-24

**Authors:** Jian Fan, X. Zhang, Y. Luo, GW. You, WK. Ng, YF Yang

**Affiliations:** 1 Department of Orthopedics, Tongji Hospital, Tongji University, Shanghai, 200065 China; 2 Department of Orthopedics, Tongji Hospital, Tongji University, Shanghai, China

**Keywords:** Tibiotalocalcaneal (TTC), Arthrodesis, Lateral approach, Reverse PHILOS plate, Cannulated screws

## Abstract

**Background:**

Tibiotalocalcaneal arthrodesis is most common and effective surgical treatment for severe hindfoot pathology, but the fusion rate is often lower than the ordinary tibiotalar arthrodesis because of the more serious joint disease associated with obvious deformity and osteoporosis. Recent literature describe tibiotalocalcaneal arthrodesis with reverse PHILOS plate with good clinical outcome result, though some patients non-union, due to eccentric force of the plate may be hidden. The purpose of this study was to evaluate clinical outcome of the lateral approach for tibiotalocalcaneal (TTC) arthrodesis with reverse PHILOS Plate and medial cannulated screw.

**Methods:**

Between Jun, 2013 to April, 2015 12 patient with hindfoot pathology had TTC arthrodesis with a reverse PHILOS plate with medial cannulated screw through a lateral approach with resection of the distal fibula and bone graft. Perioperatively observe for wound and neurovascular status. Patients were follow-up from post-operative 1, 3, 6 and12 months, to observation of wound healing, ankle pain, subtalar Joint Fusion, internal fixation and ankle function. Ankle function were scored according to the American Orthopaedic Foot and Ankle Society(AOFAS) Ankle-Hindfoot Scale system.

**Results:**

Twelve ankle fusion all patient follow-up, with mean time to surgery 18.6 months (12-36 month). No cases infection and issue necrosis; one patient complaint of lateral foot numbness we observe and follow-up was spontaneously recovery after 3 months. After 3 months of operation, no obvious pain of ankle joint and internal fixations loose were found. Almost fusion and good axial alignment of TTC joint also were found by X-ray and CT examination. After final fellow-up of each case, no case complain of pain of ankle joint, good fusion and axial alignment of TTC joint were also all found through Terminology. The mean American Orthopaedic Foot and Ankle society (AOFAS) score average was 77.5.

**Conclusion:**

TTC arthrodesis with reverse PHILOS Plate and medial cannulated screw have advantages of clear incision, effective bone orthopaedic and graft fully secure, stable internal fixation, high fusion rate and less complications, can effectively correct deformities, alleviate hindfoot pain and improve function, and is an effective method of treatment of after severe hindfoot disease.

**Trial registration:**

This trial is registered on ClinicalTrials.gov with reference number: ID: NCT02977910. Registered 26 Nov 2016, retrospectively registered.

## Background

Neglected fracture talus, talar avascular necrosis after fixation and severe inflammation ankle arthritis, may progresses to end-stage ankle arthrosis, often in tibiotalar joint and subtalar joint osteoarthritis accompanied by varus or valgus plantar flexion deformity. Patients presented with persistent foot pain, dysfunction, affected normal work and daily life activity. The goal of surgical intervention is stable fixation, pain-free fusion, correction of deformity and improve the function. Kim et al. reported that total ankle replacement combined with subtalar arthrodesis preserve hindfoot function, good clinical outcome in short tearm follow-up [1], however, there was limitation due to strict surgical indications and high demanding of surgical skills, especially patients associated with hindfoot deformity and need to observe long-term result. Tibiotalocalcaneal arthrodesis was most common surgical treatment for hindfoot pathology, its curative effect and reliable method with good result [2]. Until now method of fixation still controversia [3–6], the most widely used internal fixation for ankle arthrodesis is crossed lag screw, retrograde intramedullary nail and the blade plate [7–9]. Crossed lag screw fixation, biomechanic axial compression force and less soft tissue demage, but in patients with osteoporotic bone, lack of stability, risk of implant loosening, low fusion rate, difficulty correct varus and valgus deformity. Retrograde intramedullary nailing has biomechanical advantage, but the higher technical demanding, the insertion point must be accurate, even in the operation to calcaneal shift, or sometime cortical bone blocking can occur and tibiotalar joint displacement cause malalignment [10]. Literature reported that ankle arthrodesis with Blade plate satisfactory curative effect, even in osteoporosis patients also have a high fusion rate [11]. Complications seen with blade plate fixation include breakage of the plate and deep infection, which may require IV antibiotics and removal of the hardware. Disadvantages of the technique are related to prominence of the plate when it is placed anteriorly or laterally, which can lead to local irritation and need for subsequent removal of the plate.

Ahmad et al. are first reported applied locking plate for tibiotalocalcaneal arthrodesis, biomechanical stability, rigidity and good fusion rate with excellent result [12, 13], some patients non-union, due to eccentric force of the plate may be hidden [14]. We performed reverse PHILOS locking plate with medial cannulated screw for TTC arthrodesis, for medial lag screw can compress medial column gap and able to achieve compression lateral column at the same time, to increase the fusion rate together with lateral locking plate especially for some cases with high risk of non-union including osteoporosis and obvious deformity.

## Methods

### Patients

The TTC arthrodeses with a reverse PHILOS locking plate were done from June, 2013, through april, 2015, 7 male, 5 female; age 36–62, mean age 43.3; ankle hindfoot pathology include chronic ankle dislocation 5 cases, neglected talus fracture with ankle disclocation 4 cases, talar osteonecrosis 3 cases, ankle varus deformity 5 cases, ankle valgu derformity 2 cases, foot drop 5 cases; patient ankle range of motion plantarflexion 0°-25°, dorsifexion 10–0°, 1 cases stiffness .All Patient presented with hindfoot swelling, difficulty ambulating. Peroperative assessment for severity of hindfoot pathology, lower limb alignment and related joint arthritis with ankle x-ray AP view and lateral view and CT scan. twenlve patient AOFAS mean 35. Detail See Table 1.

**Table 1:** General information of patients and clinical outcome

No	age	Diagnosis	Associated deformity	Pre-operative AOFAS score	Healing time (PO,M)	Last follow-up AOFAS score
1	35	talar osteonecrosis	Varus deformity	25	3.2	78
2	41	chronic talus fracture with ankle disclocation	Hindfoot equinus	32	2.9	83
3	48	chronic talus fracture with ankle disclocation	Varus deformity	40	3.0	72
4	22	talar osteonecrosis	Hindfoot equinus	38	2.5	79
5	62	Chronic ankle dislocation	Valgus deformity	29	2.8	82
6	52	chronic talus fracture with ankle disclocation	Varus deformity	30	3.2	86
7	29	talar osteonecrosis	Hindfoot equinus	51	2.6	70
8	57	Chronic ankle dislocation	Varus deformity	32	2.4	82
9	33	chronic talus fracture with ankle disclocation	Hindfoot equinus	27	2.9	81
10	58	Chronic ankle dislocation	Varus deformity	31	3.0	73
11	59	Chronic ankle dislocation	Valgus deformity	40	3.0	69
12	24	chronic talus fracture with ankle disclocation	Hindfoot equinus	45	3.0	75

### Surgical techniques

**Fig. 1:**
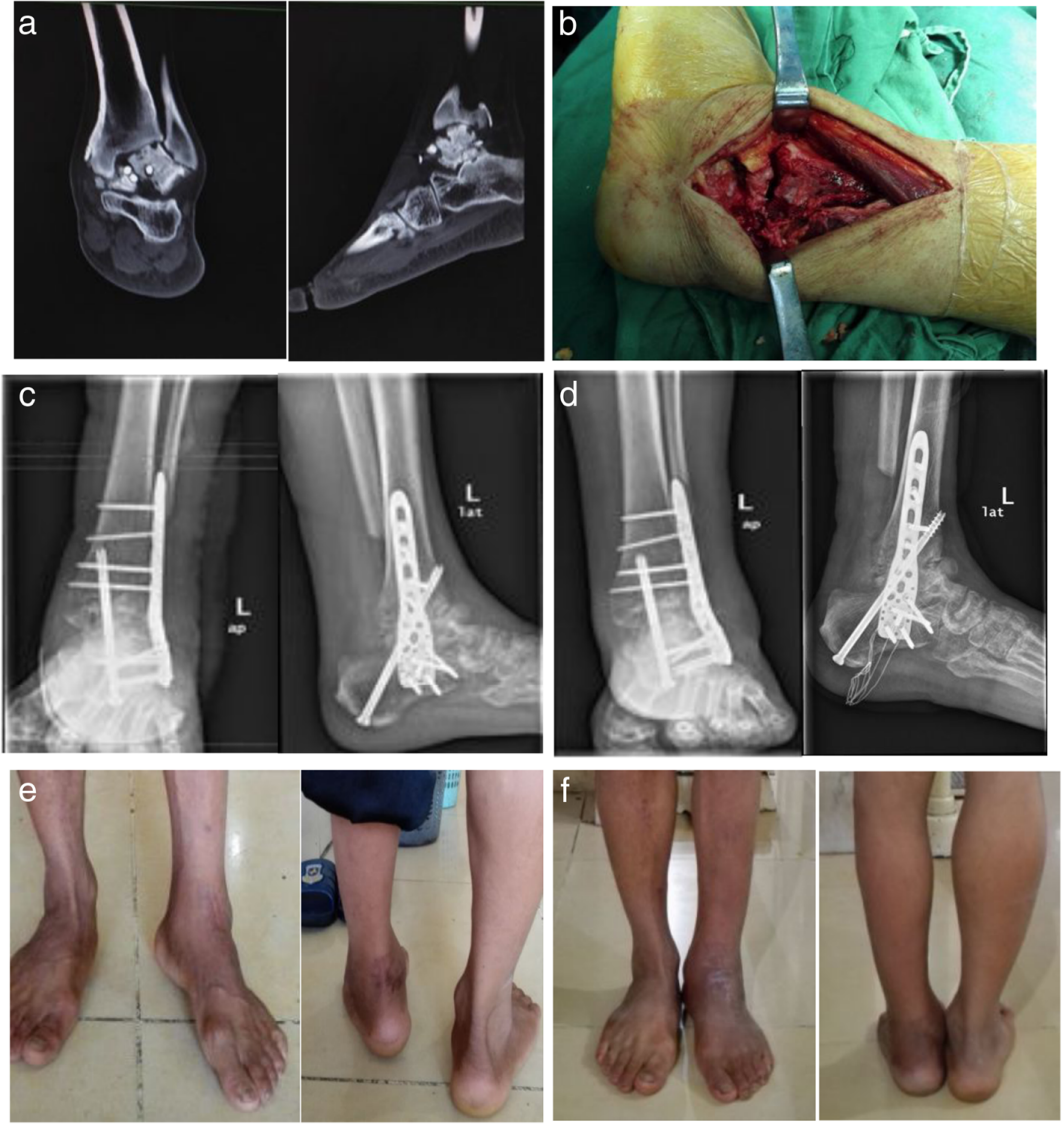
The male case, 57 years old, with left talus necrosis after internal fixation of fracture and dislocation, was treated with TTCA. **a** Preoperative CT showed avasculanecrosis of talus dislocation with old fracture and internal fixation. **b** Through the lateral incision, tibiotalar and subtalar joint were exposed after fibular was cut 7-8 cm above the distal fibula. **c** Postoperative X-ray. **d** X-ray 12 months after operation. **e** Left ankle shows obvious inversion deformity before operation. **f** Left ankle shows nearly normal 12 months after operation

Patient supine position, under general anaesthesia or spinal epidural anesthesia, clean and drapped, applied tourniquet. A curvilinear lateral incision made from 10 cm above the ankle joint extending towards the 1 cm below tip of fibula. The fibula was cut with Oscillating Saw about 7–8 cm proximal to the ankle joint, morselized, and used as a bone graft, then expose distal tibia, for tibiotalocalcaneal joint, talus, subtalar joint and part of calcaneus. Removal arthritic articular cartilage surface together with the subchondral plate curettage until bleeding bone was seen, bone cut and release soft tissue to correct any deformity and aligment. Articular surface drill with 1.5 mm Kirschner wire. If the tibiotalar joint space narrowing, difficult exposure, can make a small incision about 2 cm in medial malleolus, curettage and removal of articular cartilage. In complex valgus or varus deformity, we can performed wedge osteotomy distal of talus to correct derformity, if there was severe deformity with osteoporotic bone, osteotomy wedging fill in with harvested tricortical iliac crest bone graft. At the same time tibiotalocalcaneal and subtalar joint space insert morselized fibula graft. In old fracture talus with dislocation of ankle joint should reduce the talus and maximal tibiotalar joint contact surface, increase fusion rate and preserve the limb length. Ankle fixed on plantar flexion 0 degrees, 0–5 degrees valgus and external rotation of 5–10 degree, 1st Kirschner wires inserted from lateral 1/3 plantar region, aim 35–40 degree, second Kirschner wires inserted 3 cm above tip of medial malleolus, third Kirschner wires inserted through heel from calcaneus upward to talus until distal tibia level, check and confirm alignment with X-ray image intensifiers, we using a 5.0 mm cannulated drill bit and self-tapping, 6.5 mm cannulated cancellous screws partially threaded was placed from posteroinferior to anterosuperior across the calcaneus, talus, and distal tibia, compressing both the ankle and subtalar joints meanwhile drawing the other two Kirschner wires. PHILOS plate reserve and applied to tibia bone, proximally fixed to the calcaneus and talus with locking screw with multiple planes, then insert distal screw (Fig. 1).

### Postoperative care

Post-operative wound care, to complete 24 h antibiotics to prevent infection. To observe wound healing and neurovascular status. Day 1 rehabilitation, toe Range of motion exercise, post-operative 1 month, 3 month, 6 month and 12 month follow-up observe for pain, complication, radiographs were used to assess fusion, internal fixation and aligment. To evaluate AOFAS score, once radiographically show evidence of bony healing allow gradually weight bearing 25% of body weight with air cast, it may off once clinically and radiograhically show fracture healing.

## Results

Post-operative no patient defaulted follow-up, 12 month follow-up was obtain, mean follow-up time 18.6 month (12-36 month). Post-operative 2 weeks all wound healing, no cases of infection; 1 patient presented with lateral foot numbness, we follow-up and observe, symptom resolve after 3 month; post-operative 3 month patient no pain, radiographic show bone union, no sign of implant failure; post-operative 12 month all patient achieved a stable pseudarthrosis, which was painless and uninfected at last follow-up. The average AOFAS score improved from 35 points preoperatively to 77 points postoperatively (Table 1).

## Discussion

The main component of hindfoot include tibiotalar joint and subtalar joint. Tibiotalocalcaneal joint pathology may cause chronic pain, deformity, affected ambulating and daily life activities. Severe arthritis of ankle, avascular osteonecrosis, malunion of talus hindfoot pathology associated with varus or valgus plantar flexion deformity, poor result in conservative treatment, tibiotalocalcaneal arthrodesis is effective treatment with good clinical outcome [15, 16]. Because of the joint disease for tibiotalocalcaneal arthrodesis is more serious, and are mostly associated with osteoporosis, the fusion rate is often lower than the ordinary tibiotalar arthrodesis, therefore, a safe and reliable of tibial subtalar joint fusion operative technique is attraction orthopaedic surgeon. Cross cannulated screw and retrograde nail is most common in tibiotalocalcaneal arthrodesis, but cannulated screw biomechanical stability insufficient, retrograde nail high demanding on surgical skill and implant irritable. Modified proximal humerus locking plate can reduce complication, increase rate of union and fusion. In china, plate for tibiotalocalcaneal arthrodesis is uncommon, recent literature describe tibiotalocalcaneal arthrodesis with reverse PHILOS plate with good fusion rate, less complication, good clinical outcome result. Study does have some inherent limitations, including length of follow-up and sample size [17].

Our study, retrospective study, and non-comparative, evaluated clinical outcome of the lateral approach for TTC arthrodesis with reverse PHILOS Plate and medial cannulated screw, it is more advantage if compared with other method. For reason with: (1) locking plate is stables and rigid fixation, achieve stable in fusion. The lateral locking plate fixation is eccentric fixation, and it is inevitable to have an impact on the stability of the partial medial deformity correction and severe osteoporosis patients. We demonstrated reverse PHILOS locking plate for TTC arthrodesis with additional 6.5 mm cannulated medial screw to converted eccentric force to centre axis compression force, compress medial gap, increase axis compression force, and increase the fusion rate (2). Medial screw compression and reduce the medial gap. Tibiotalocalcaneal joint temporary hold with K-wire then fix with single locking plate TTC arthrodesis technique may have gap and unstable with tibiotalocalcaneal joint fusion may cause implant failed. This study PHILOS plate and pack with bone graft after medial cannulated screw improve successful rate of fusion. In our study all patient TTC arthrodesis is union with good AOFAS score (3). In this study, lateral approach is great anatomical exposure for TTC arthrodesis for reverse PHILOS plate fixation, bone graft packing area and less surgical skill demanding require. After fibular reserted can fully expose with self-retainer retractor the tibiotalar joint, talus and subtalar joint fusion to avoid compromise surgeon procedure quality (4). Resection of fibula, distal fibula bone contain of cancellous bone can be used as bone graft, it may avoid iliac crest autologue bone graft. Plate located at laterally of tibia localised soft tissue not tense, risk of skin necrosis and surgical site infection is rare.

Philos plate is absolute stability for tibiotalocalcaneal arthrodesis with a good clinical outcome result in this study, although it is not a special design implant for tibiotalocalcaneal arthrodesis, according to biomachanic of locking plate more stiffness and rigidity to the arthrodesis can prevent collapse. Force of load sharing more are located in the anterior part of the calcaneus, a new plate design is necessary for biomachanic. Even though this study have good clinical outcome, In view of ethical problems therefore, it is necessary to design a special locking plate according to plasticity, anatomy and screw distribution according to biomachanic principle to improve the stability and fusion rate of TTC arthrodesis. After distal fibula resertion will compromise ankle joint micro-movement and fibula weight bearing, strict indication for end stage ankle arthritis is necessary. This study also does have some inherent limitations, including long team follow-up, size of study, comparisons between group of patients and biomechanical test study in future.

## Conclusions

TTC arthrodesis with reverse PHILOS Plate and medial cannulated screw have advantages of clear incison, effective bone orthopaedic and graft fully secure, stable internal fixation, high fusion rate and less complications.This study shows that good clinical outcome with less demanding of surgical technique with reverse PHILOS plate and medial screw for TTC fusion.

## Abbreviations

AOFAS: American orthopaedic foot & ankle society
PHILOS: Proximal humerus internal locking system
TTC: Tibiotalocalcaneal
